# Identification of mechanisms enabling integrated care for patients with chronic diseases: a literature review

**DOI:** 10.5334/ijic.1127

**Published:** 2014-07-21

**Authors:** Denise van der Klauw, Hanneke Molema, Liset Grooten, Hubertus Vrijhoef

**Affiliations:** TNO, P.O. 3005, 2301 DA Leiden, The Netherlands; TNO, P.O. 3005, 2301 DA Leiden, The Netherlands; TNO, P.O. 3005, 2301 DA Leiden, The Netherlands; MD3, National University of Singapore, 16 Medical Drive, 117597 Singapore

**Keywords:** delivery of health care, integrated, integrated health-care systems, health-care reform, classification, literature review, models organisational

## Abstract

**Introduction:**

Notwithstanding care for chronically ill patients requires a shift towards care that is well coordinated and focused on prevention and self-care, the concept of integrated care lacks specificity and clarity. This article presents a literature review to identify mechanisms for achieving integrated care objectives.

**Theory and methods:**

Existing models often present a large variety of dimensions, archetypes and categories of integration without specifying them. Models and programmes describing integrated care for chronic diseases were reviewed. Data were extracted related to objectives and clusters of mechanisms of integration.

**Results:**

Thirty-four studies presented four objectives: functional, organisational, professional and service integration. We categorised approaches and interventions to achieve these objectives by strategy and clusters of ‘mechanisms of integration’: degree, patient centredness and normative aspects.

**Conclusions and discussion:**

The clarification of mechanisms to achieve objectives of integrated care as presented may be used as starting point for the development and refinement of integrated care programmes, including methodological grounding of their evaluation. Given that most studies reviewed lack both empirical data and descriptions of the methods used, future research needs to close these gaps. Validation of the findings by a large panel of experts is suggested as recommendation to work towards a grounded framework.

## Introduction

Developed countries increasingly recognise the need for a change in health delivery systems, towards a system that provides improved patient experiences of care, improved health of populations and reduced costs of health care per capita (‘Triple Aim’) [1]. The most important means to realise this end is by developing a system including a network of organisations that increasingly arranges equitable, comprehensive, integrated and continuous health services [2]. Such a system is indicated as ‘integrated care’ and can be described as:

a coherent set of methods and models on the funding, administrative, organizational, service delivery and clinical levels designed to create connectivity, alignment and collaboration within and between the cure and care sectors [3].

Notwithstanding integrated care receiving worldwide attention in improving health-care delivery, various definitions of this concept and its constitutive elements exist. Managed care, continuity of care, transitional care and disease management are examples which integrated care is equated with. The commonly used definitions are often vague and confusing [4]. According to Nolte and McKee, this confusion reflects the polymorphous nature of a concept that is applied from several disciplinary and professional perspectives and associated with diverse objectives [5].

This lack of specificity and clarity highly impedes successful application and expansion of integrated care systems in practice [4, 6]. Further, this gap makes understanding difficult how integrated care systems can deliver greater value for the resources devoted to health-care delivery. Whereas some attempts have been made to shed light on elements of and conditions for integrated care systems, not yet have the mechanisms that enable the achievement of the objectives for why integrated care is being pursued, been systematically studied in the scientific literature. The aim of our study is to aggregate scientific articles that provide a description and/or a refinement of an integrated care model or programme, and to identify the state of current literature about mechanisms that can or have been used to achieve certain objectives. The paper concludes with possible benefits of the provided overview for different stakeholders and provides recommendations for further research.

## Theory and methods

### Theoretical background

The expanding literature about models and descriptions of integrated care provides a useful and rich base to enhance the understanding of integrated care. Although their outlooks are rich and varied, available research strands have certain shortcomings. First and foremost, existing theories often present a large variety of dimensions, archetypes, classifications and key elements of integration, which they lack to specify [5]. Moreover, they differentiate between ‘types’ of integration [7, 8]; ‘dimensions’ of integration, such as type, breath, degree and process [5]; ‘archetypes’ of integration, i.e. foci of integration, types, levels, breadth and degree [4]; ‘classifications’ in objects and components of integrated care [9]; and key elements of integrated care for specialised segments, such as primary care [10].

Although their scope and language may differ, they broadly agree on the major objectives for why integrated care is being pursued. These major objectives can be summarised as:

- functional integration: describes the extent to which back office and key support functions are coordinated, as well as the alignment of (financial) incentives;
- organisational integration: aims at the relationships between health-care organisations in a health network, such as ownership, contractual arrangements and alliances among organisations;
- professional integration: describes provider relationships within and between organisations;
- service integration (also referred to as clinical integration): refers to the coordination of services and the integration of care in a single process across time, place and discipline to maximise the value of services delivered to patients.

We left breadth and level of integration out as distinctive elements in this summary, since they indicate the application or development state of these objectives of integration and are not an objective in itself [4, 5, 7–9].

Second, although existing theoretical discourses provide a comprehensive insight in the essential elements of a health-care system, they lack practical guidance to illuminate the concept of integrated care by describing mechanisms to achieve integrated care. These important shortcomings are unfortunate because they enforce that insights on mechanisms about how integrated care can lead towards improved outcomes, such as degree of integration, aspects of patient-centredness and normative, or ‘soft’ aspects of integration [4–10], remain mainly on a high and abstract level. The result is a jumble of stand-alone integrated care initiatives in practice. Whereas these initiatives mostly share a common goal (i.e. improved patient experiences of care, improved health of populations and reduced costs of health care per capita) and objective, they lack a common, specified base, which impedes comparison and structural evaluation of integrated care initiatives and benchmarking of such initiatives. In addition, the lack of insight leaves health-care professionals, researchers as well as policy-makers guessing about which integrated care mechanisms have best chances of success given a certain context.

The current literature review may assist in resolving the organisational and professional uncertainty, which is currently apparent. This paper aims to provide a clear insight into which mechanisms are related to achieving the summarised objectives of integrated care. This brings us closer to understanding how integrated care can lead to better health, better patients’ experiences and lower costs.

## Methods

A literature review was performed. The focus of the review was narrowed down to common chronic diseases (i.e. pulmonary diseases, coronary heart diseases, diabetes, depression and cancer and chronic illness in general), rather than the broad spectrum of subjects of integrated care, since common chronic diseases make up the largest burden for health-care systems of OECD countries [11]. Further, given the disease-specific orientation of most current integrated care initiatives, this focus reflects the scientific literature most realistically.

### Search strategy

We developed a search strategy that included key terms from the existing theoretical discourses and related items to the concept of integrated care. Search strings were set up consisting of a selection of 10 search terms (‘case management’, ‘care coordination’, ‘community health services’, ‘comprehensive health care’, ‘continuity of patient care’, ‘disease management’, ‘health-care reform’, ‘managed care programmes’, ‘patient participation’ and ‘self-care’) and 6 combination terms (‘classification’, ‘organisational models’, ‘terminology’, ‘chronic disease’, ‘integrated delivery of health care’ and ‘quality assurance’; see Appendix A for full electronic search strategy).

We searched PubMed for articles published between 1990 and 2012. To avoid misinterpretation, articles in languages other than Dutch or English were excluded. Other exclusion criteria were:

- articles that did not focus on integrated care as key subject (e.g. studies about post-rape care, pain management and drug abuse);
- articles not about most common chronic diseases (e.g. Multiple Sclerosis, renal diseases and human immunodeficiency virus);
- articles describing programmes or models for specific target groups (e.g. mother and child care, adolescent care, palliative care, acute care, homeless people, prisoners);
- articles about non-OECD countries; and
- articles solely describing methods of care delivery (e.g. Planetree/Magnet).

The search results, i.e. titles, abstracts and articles, were independently assessed by two researchers (LG and DvdK). In case of disagreement, a third researcher (HV) acted as referee.

### Data analysis

For every included article, it was determined whether it was ‘based on empirical data’, ‘not based on empirical data’ or ‘narrative review’ (i.e. discussing or refining an existing model of integrated care). Additionally, we briefly described the methodological foundation of each article. Included articles were analysed using a deductive approach. Starting point were the four major objectives of integrated care, as described above:

1. functional integration;
2. organisational integration;
3. professional integration; and
4. service integration.

To be able to identify approaches or interventions that are described in literature as mechanisms for achieving these objectives, we categorised mechanisms in three clusters:

1. the *degree* in which integration efforts between professionals and organisation are effectuated was presented by Leutz [12], whose classification (linkage, coordination, integration) is still leading in the models of Kodner, Nolte and McKee and Valentijn et al. [4, 5, 10]. To reach the degree of integration, one has to establish lower degrees first. Further, the highest level is not required in every context.
2. *patient-centredness* was not specifically present in the overviews of Fulop et al., Nolte and McKee, and Kodner and Valentijn [4, 5, 7, 10], but was recently addressed by Singer et al. [9]. They defined patient-centredness as the manner in which care is designed to meet patients’ needs and preferences. Singer et al. addressed this mechanism as an independent objective because of the importance of the patients’ perspective in the ambitions to deliver improved quality by meeting the needs of patients [9]. Nevertheless, it may be argued that patient-centredness may contribute as a mechanism towards all objectives. Ideally, the degree of integration corresponds to the social and health needs and preferences of individuals of a certain population [13];
3. *normative aspects* are about the values and culture of integration. Valentijn et al. emphasised that these aspects may act as a ‘common frame of reference’ that bind an integrated system [10]. Kodner defines normative aspects as a separate type of integration [4], whereas Nolte and McKee address these aspects as a ‘process’ of integration [5]. Given that normative aspects actually run through functional, organisational, professional and service integration, as Valentijn et al. emphasised [10], we consider these as a cluster of mechanisms rather than an objective of integration.

Next to these three clusters, we identified the *strategy* followed in the reviewed publications. Kodner mainly regards the strategy as the target populations, such as patients with complex illnesses, vulnerable subgroups as frail elderly or the entire community [4]. Singer et al. relate strategy to the setting in which integration is coordinated, i.e. targeted to one organisation, multiple organisations or the population as a whole [9]. Valentijn et al. make a distinction to the macro-, meso- and micro-level, which each has a specific objective of integration, according to their framework [10].

Descriptions in literature concerning objectives and clusters of mechanisms were marked and listed in MS Excel. After identifying, the articles were thoroughly analysed to identify labels in the descriptions of integrated care presented in the articles. Data were first extracted on the objectives that were pursued in an article. Second, we identified which mechanisms were distinguished in the articles to achieve a certain objective. Since we searched for descriptions or refinements of integrated care models or programmes, we were only able to identify descriptions of mechanisms and were unable to relate them to the success of a certain approach.

## Results

### Summary of the search results

**Figure 1.**
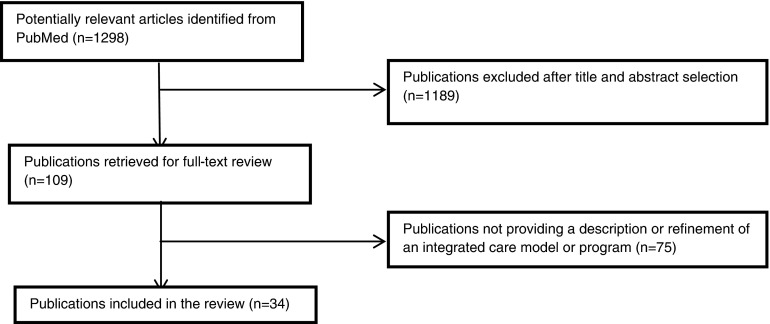
Results of search strategy

The electronic search selected 1,298 titles of which 820 abstracts were retrieved for appraisal (Figure 1). This led to exclusion of 737 abstracts, including 154 untraceable articles. Of the remaining 109 publications, 75 articles were excluded because the full-text article was not traceable (*n* = 39) or the article did not meet the inclusion criteria (*n* = 36). The final review included 34 studies for analyses [4, 14–46]. Two sessions were held to discuss articles with the referee.

Of the selected studies, only five were based on empirical data [15, 17, 31, 33, 39]. Of the remaining 29 studies, 19 were narrative reviews [4, 14, 18, 22–24, 28, 30, 34, 36–38, 40–46]. Overall, the 29 studies without empirical data often lacked to describe the methods properly and did not provide full information about data collection and/or the process of result synthesis.

### Mechanisms of integrated care

With the aim to identify and converge insights in the mechanisms to achieve objectives of integration, first, the selected articles were divided according to the objective of integration (Table 1).

Six articles described only 1 objective of integration [4,17,28,34,35,36], 13 articles presented 2 objectives [14, 19, 21, 22, 26, 27, 29, 30, 38, 40], 8 publications presented 3 objectives [15, 20, 23, 31, 32, 43, 45, 46] and in another 7 articles all 4 objectives of integration were identified [16, 18, 24, 25, 33, 37, 39].

All reviewed publications described a strategy for focus. Thirteen publications focused on chronic patients in general [4, 15, 18, 24, 31, 35–37, 40–44] and five on the whole population [28, 32, 39, 45, 46]. The other 16 papers could be divided into studies addressing disease-specific patient groups [14, 17, 19–21, 23, 26, 27, 33], more generic groups like frail elderly [16, 25] and patients with multi-morbidity [38], and patient populations based on geographical boundaries [29] or the specific setting of the hospital [22, 30, 34].

A majority of publications presented a single cluster of mechanisms (degree, patient-centredness or normative aspects) as the primary enabler of achieving a certain objective related to in their descriptions (70% of the publications aimed at functional integration, 60% of the publications worked towards organisational integration, 83% of the publications described professional integration and 52% of the publications focused on service integration). A multiple mechanism approach, thus a combination of degree, patient-centredness and/or normative aspects, was used in 30% of the publications aiming at functional integration, in 40% of the publications aiming at organisational integration, in 17% of the publications describing a model towards professional integration and in 48% of the publications that work towards service integration (Table 2). The findings of the literature review are summarised in Table 3.

### Functional integration

Ten of the publications describing a functional objective of integration followed a strategy on chronic patients [4, 15, 18, 24, 31, 35, 37, 42–44]. Another five publications addressed disease-specific patient groups [14, 20, 21, 23, 33], while the other publications focused on broader groups, such as elderly [16, 25], the population in general [39, 45] and hospitalised patients [22]. An important aspect of the *degree* of integration was the use of clinical information and communication technologies, as was shown in nine articles [14, 15, 18, 20, 24, 25, 31, 33, 35]. The articles emphasised information and communication technologies as a mechanism on which integration efforts should focus because they enable support for coordination of care and information sharing between professionals and between organisations (‘linkage’). They presented reminder systems, systems for information sharing and decision-making, and systems to provide an overview of the patient population. In a similar manner, articles discussed the use of information and communication technologies in facilitating *patient-centredness*, e.g. as a tool to remind care providers for prevention-screening interventions and follow-up of patients [23, 35, 37]. In addition, the reviewed articles described information and communication technologies that enable self-management support for patients [4, 14, 20, 22, 43]. Financial compensation and incentives were addressed in the literature as part of the *normative aspects* of functional integration. Nine publications, especially, related financial incentives to performance measurements to promote effective chronic care practices [15, 16, 21, 25, 32, 37, 39, 42, 45].

### Organisational integration

Eight publications describing the organisational integration followed a strategy towards chronic patients [4,15,18,24,37,40,43,44]. The other publications addressed the whole population [39, 45, 46] or subgroups as frail elderly [16, 25] or hospitalised patients [30]. One article focused on a specific disease [33]. The *degree* of organisational integration was based on three aspects:

- formal connections between organisations;
- connection between health care and community services; and
- methods and tools for organisational integration.

Seven articles described connections between health-care organisation and the community, such as the workplace, sport centres, peer support/support groups, educational programmes and prevention services [15, 16, 18, 24, 33, 43, 45]. Examples given of methods and tools to achieve connections were umbrella organisations [25] and mergers and strategic alliances [4, 39]. Concerning *patient-centredness*, the transitions between community, professionals and others were emphasised [46]. Care pathways were used to assure this continuity. According to the literature, care pathways should include expected patient outcomes related to multidisciplinary care processes at key time intervals and should be able to adapt to different needs of patients [25, 30, 40, 44, 45]. From a *normative aspect* on integration, organisational leadership, culture, policy and responsibility were mentioned by six publications [16, 18, 25, 33, 37, 39]. They emphasised the need for a shared vision and disposition that reflects the intentions for integration from the organisation.

### Professional integration

Publications pursuing professional integration followed most often a strategy focused on chronic patients [18, 24, 31, 36, 37, 40, 41, 43], specific diseases [19, 20, 23, 26, 27, 33] or the whole population [28, 32, 39, 46]. Other articles were aimed at frail elderly [16, 25], hospitalised patients [30, 34], patients with multi-morbidity [38] or a patient population demarcated by geographical boundaries [29]. The *degree* of integration depended on the strength of connection within multi- or interdisciplinary teams, which was described by seven articles, varying from linkage to coordination and to ‘full’ integration [16, 20, 23, 25, 26, 37, 46]. According to the selected articles, the composition of the teams might vary and could include health and social professionals, nurse (case) managers, mid-level professionals (physician assistants, clinical pharmacist) and pharmacists [16, 19, 20, 23, 27, 30, 33, 36]. For coordination as part of professional integration, clear assignment of key roles in a multidisciplinary team was identified in 11 articles [18, 20, 28, 31, 32, 37–41, 43]. Mechanisms regarding *patient-centredness* from the perspective of professional integration were covered in the selected publications by information sharing and meetings. These were considered to ensure continuity and correspondence of services for the patient [24–26, 29]. Three articles specifically identified mechanisms in the cluster of *normative aspects* working towards professional integration [34, 38, 39]. One article emphasised the maintenance of autonomy of team members, as another focused on motivation, values and morale as a driver of integration.

### Service integration

The majority of publications describing service integration followed a strategy on chronic patients [15, 18, 24, 31, 37, 41–44] or disease-specific patient groups [14, 17, 19–21, 23, 26, 27, 33]. Four articles described a strategy for the whole population [32, 39, 45, 46] and two for frail elderly [16, 25]. Additionally, there was one article describing a strategy for hospitals [22], one for patients with multi-morbidity [38] and one for patient groups within a specific geographical area [29] that described mechanisms towards service integration. The *degree* of integration depended on the use of evidence-based guidelines because they standardise care and decision support across different care providers and sites [14–16, 18, 23, 24, 29, 37, 39, 41, 42, 44, 45]. The degree of service integration was also related to mechanisms describing the build infrastructure. The selected articles presented three variations on how build infrastructure can contribute to integrated care. First, two articles described geographically co-locating disciplines and departments as an example of this object of integration [22, 31]. Second, a more intensive form of integration of infrastructure was described in an article about a centralised diabetes clinic operating under a single roof to provide integrated care [20]. Third, coordinated patient visits to one site scheduled in a continuous series of appointments with various professionals was another example of build infrastructure integration [17, 37]. The literature presented three integration mechanisms related to the cluster of *patient-centredness* that worked towards this objective of integration: collaborative care planning, offering self-management support and involving the family. One article, based on experiences in mental health care, studied the use of a common care plan, which included a motivational tool, measurement of outcomes over time, a communication tool, a record of individual patient information and patients’ self-defined problem and goals [15]. Either, collaborative goal setting and problem solving between patients and clinicians were addressed in five articles [20, 23, 26, 37, 43, 44]. Self-management support followed from collaborative care planning and was presented as help for patients and their families to manage their chronic conditions [14, 18, 19, 27, 32, 41, 43, 45, 44, 46]. Amongst others, education was described as important part of self-management [18, 22, 23, 25, 33, 38]. How education was provided differed from individual to group sessions and from face-to-face to digital learning programmes. Two publications especially stressed the role of the family in this process of collaborative care planning and self-management [14, 16]. To facilitate service integration, a paradigm shift was regarded a required *normative aspect*. Again, education was described as an enabler [14]. In addition, publications described that members of the health team should be aware of, have access to, and are educated in the most up-to-date clinical care information for providing care to their chronically ill patients [14, 15, 19–21, 33, 37, 44, 45].

## Discussion

This literature review presents the mechanisms to achieve major objectives of integrated care, as they were reported in descriptions or refinements of integrated care models or programmes. The major objectives of integrated care, which were leading in this review, were deducted from theoretical frameworks. These frameworks currently lack specificity on actual approaches that can be or have been used to integrate care. By identifying the state-of-the-art literature on mechanisms and relating them to the objectives of integrated care from a broad perspective on chronic diseases, this review goes beyond current theoretical frameworks.

Our review shows that, especially for the objectives of functional and organisational integration, most reviewed studies followed a strategy for chronic patients in general. Strategies for patients with multi-morbidity and for patient populations bounded by a geographical area were a scarce in our review and were only described in publications aiming at professional or service integration.

The majority of reviewed publications present a single cluster of mechanisms to achieve an objective, although combinations between degree, patient-centredness and normative mechanisms are made. This is especially to work towards the objective of organisational and service integration. However, in practice, multiple mechanisms may be utilised by practitioners, which were not described in the reviewed publications, such as Human Resource Management or change management activities. Therefore, more detailed descriptions of integration programmes and strategies are demanded to be able to further unravel the concept of integrated care. Regarding the mechanisms of integrated care that are reported in current literature, our literature search shows that the existing mechanisms to achieve integrated care are rich and varied as well as thin, both in their empirical foundation and in their description of the mechanisms used. The majority of studies identified in this paper are not based on empirical data and lack a description of the methods used. As a result, information about the data collection process and the synthesis of results was missing and the methodological quality of these articles is questionable. This lack of empirical evidence restricted our review to the descriptions of the mechanisms used, since we were unable to provide any evidence on the effectiveness of strategies. The paucity of studies describing the applied methods can indicate that researchers are developing models of integrated care without systematically observing existing theories and/or best practices. We consider these thin descriptions or narrow approaches as an important limitation to expand successful integrated care strategies and to learn from the ones that were less successful. We recommend additional research on integrated care that is grounded in clear theories, of which the identified mechanisms in this literature review could serve as a starting point. Only then, we will be able to identify successful strategies that can be utilised to achieve a specific objective of integration.

Further validation of our findings with patients, practitioners, health-care insurers, policy-makers and researchers would contribute to further optimisation of the findings and the applicability of the insights in daily practice.

### Study limitations

The aim of this study was to aggregate scientific literature and identify its state, not to present a new framework or model for integrated care, since some limitations may apply. First, not all abstracts and full-text articles selected from the PubMed database were available and could as such not be included in this concise review. Second, we only searched the PubMed database. For further investigation, other databases, such as Web of Science could be used. Further, to delineate the literature search and to counteract heterogeneity between articles, the inclusion of articles was restricted to articles with a description or refinement of models of integrated care and disease management. Consequently, process and implementation studies were excluded and information on integrated care in practice could have been missed. Furthermore, future research may focus on specific mechanisms for multi-morbidity, since these were not a specific search string in our current review. A next step is to move towards a framework validated by the major stakeholders. A first review of the findings to indicate face validity was executed in interviews with two experts on integrated care at two large health-care insurance companies in the Netherlands. This small-scaled review showed that health-care insurers agreed that the identifications of mechanisms related to objectives could contribute to solving problems in purchasing care and in quality monitoring of purchased products, by clarifying terminology and classifying integrated care concepts. The interviewees recognised the objectives and clusters of mechanisms that were the point of departure for this literature review. Nevertheless, the health-care insurers remarked that integrated care in daily practice is volatile and developing. Further validation of the findings in a large panel of experts in integrated care, representing practice, policy and science is therefore recommended to develop a grounded framework. Further, we did not assess the methodological quality of selected studies in detail, given the variation in study designs and the lack of details on study methods. The latter emphasises that the methodological quality of studies was poor, which should raise attention for future research in this field, as was advocated by this review.

## Conclusion

In summary, this literature review adds to the understanding of integrated care by specifying mechanisms to achieve objectives that are pursuit to integrate care, which were previously introduced and fostered in many theoretical frameworks. Because most descriptions and refinements of models for integrated care found in this literature review lacked a empirical base, the applicability of the findings for research on effectiveness of integrated care is still restricted. Although this literature review may only reflect the current state of literature, it could be a useful starting point for the development and refinement of integrated care with stronger theoretical and methodological grounding. The findings could move towards a framework that could be used for benchmarking current or future integrated care initiatives and to identify (relations between) working mechanisms, which facilitate quality improvement of both existing initiatives and new initiatives.

## Figures and Tables

**Table 1.**
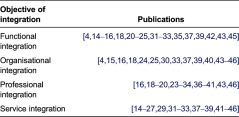
Publications per object of integration

**Table 2.**
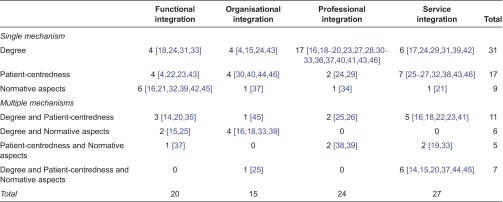
Number of studies per objective, describing a single or multiple clusters of mechanisms

**Table 3.**
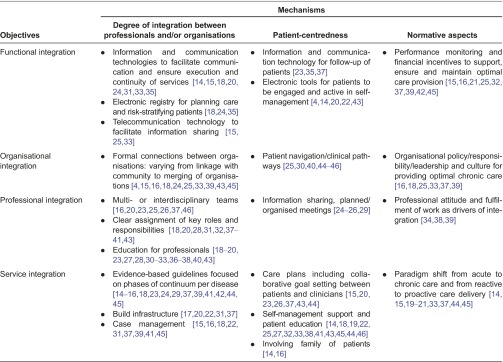
Summary of findings from the literature review

## Appendix A

The final combination of search terms was: (“community health services”[MeSH Terms] OR (care[All Fields] AND coordination[All Fields]) OR “managed care programs”[MeSH Terms] OR “comprehensive health care”[MeSH Terms] OR “disease management”[MeSH Terms] OR “health care reform”[MeSH Terms]) AND “models, organizational”[MeSH Terms] AND (“chronic disease”[MeSH Terms] OR “quality assurance, health care”[MeSH Terms] OR “delivery of health care, integrated”[MeSH Terms])
